# A Review on the Role of Small Nucleolar RNA Host Gene 6 Long Non-coding RNAs in the Carcinogenic Processes

**DOI:** 10.3389/fcell.2021.741684

**Published:** 2021-10-04

**Authors:** Soudeh Ghafouri-Fard, Tayyebeh Khoshbakht, Mohammad Taheri, Seyedpouzhia Shojaei

**Affiliations:** ^1^Department of Medical Genetics, School of Medicine, Shahid Beheshti University of Medical Sciences, Tehran, Iran; ^2^Men’s Health and Reproductive Health Research Center, Shahid Beheshti University of Medical Sciences, Tehran, Iran; ^3^Skull Base Research Center, Loghman Hakim Hospital, Shahid Beheshti University of Medical Sciences, Tehran, Iran; ^4^Department of Critical Care Medicine, Imam Hossein Medical and Educational Center, Shahid Beheshti University of Medical Sciences, Tehran, Iran

**Keywords:** SNHG6, lncRNA, cancer, biomarker, expression

## Abstract

Being located on 17q25.1, small nucleolar RNA host gene 6 (SNHG16) is a member of SNHG family of long non-coding RNAs (lncRNA) with 4 exons and 13 splice variants. This lncRNA serves as a sponge for a variety of miRNAs, namely miR-520a-3p, miR-4500, miR-146a miR-16–5p, miR-98, let-7a-5p, hsa-miR-93, miR-17-5p, miR-186, miR-302a-3p, miR-605-3p, miR-140-5p, miR-195, let-7b-5p, miR-16, miR-340, miR-1301, miR-205, miR-488, miR-1285-3p, miR-146a-5p, and miR-124-3p. This lncRNA can affect activity of TGF-β1/SMAD5, mTOR, NF-κB, Wnt, RAS/RAF/MEK/ERK and PI3K/AKT pathways. Almost all studies have reported oncogenic effect of SNHG16 in diverse cell types. Here, we explain the results of studies about the oncogenic role of SNHG16 according to three distinct sets of evidence, i.e., *in vitro*, animal, and clinical evidence.

## Introduction

Small nucleolar RNA host gene 6 (SNHG16) is a member of SNHG family of non-coding RNAs. Long non-coding RNAs (lncRNAs) are a class of transcripts that have sizes longer than 200 nt. These transcripts serve as scaffolds for establishment of different complexes of biomolecules. Moreover, the can serve as enhancers, modulators of chromatin structure and decoys for several molecules, particularly miRNAs {Zhang, 2019 #481}. Bioinformatics tools have facilitated identification of several classes of lncRNAs among them is SNHG group of lncRNAs {Li, 2020 #482}.

Being annotated as NC_000017.11, *SNHG16* gene is located on 17q25.1 and has 4 exons. Based on the Ensembl database^1^, 13 splice variants have been identified for this SNHG16 with one of them having a retained intron (ENST00000587743.1) and the rest being categorized as long non-coding RNAs (lncRNAs). These transcripts have sizes ranging from 556 nt (SNHG16-208) to 3607 nt (SNHG16-201). No protein has been recognized for any of these variants. It has been shown to be ubiquitously expressed in ovary, skin and several other tissues. This lncRNA has fundamental roles in the carcinogenesis in numerous types of tissues. Here, we summarize the results of these studies based on three distinct categories of evidence, i.e., *in vitro*, animal and clinical evidence.

## Cell Line Studies

**FIGURE 1:**
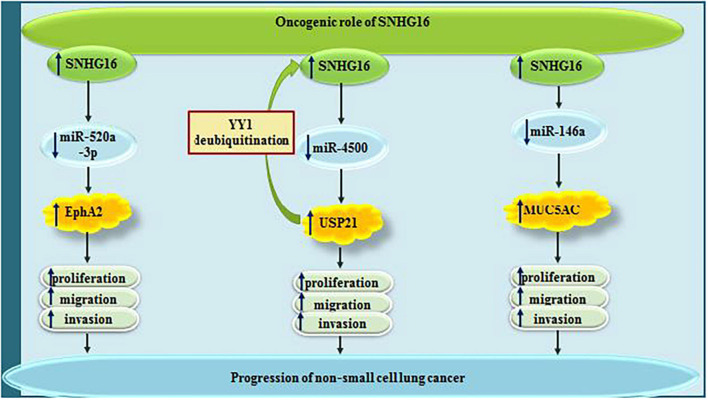
The oncogenic roles of SNHG16 in lung cancer are mainly mediated through sponging miR-520a-3p, miR-4500, and miR-146a.

Small nucleolar RNA host gene 6 has been demonstrated to be up-regulated in lung cancer cell lines, where it acts as a sponge for miR-520a-3p. Through decreasing the availability of this miRNA, SNHG16 increases expression of EphA2. SNHG16 silencing has suppressed proliferation, migratory potential and invasiveness of these cells, while stimulating cell apoptosis. Further experiments have shown the prominence of SNHG16/miR-520a-3p/EphA2 axis in the regulation of oncogenicity in lung cancer (Yu et al., 2020). Being transcriptionally regulated by YY1, SNHG16 also sequesters miR-4500 to modulate expression of the deubiquitinase USP21. USP21 can further increase expression of SNHG16 (Xu P. et al., 2020). Another experiment in lung cancer cells has identified miR-146a as the target of SNHG16, through its sequestering SNHG16 enhances proliferation, migration and invasiveness of lung cancer cells. The sponging effect of SNHG16 on this miRNA leads to over-expression of MUC5AC, a protein which accelerates metastasis and recurrence of lung cancer cells (Han et al., 2019). Figure 1 depicts the roles of SNHG16 in lung cancer which are exerted via sponging miR-520a-3p, miR-4500 and miR-146a.

**FIGURE 2:**
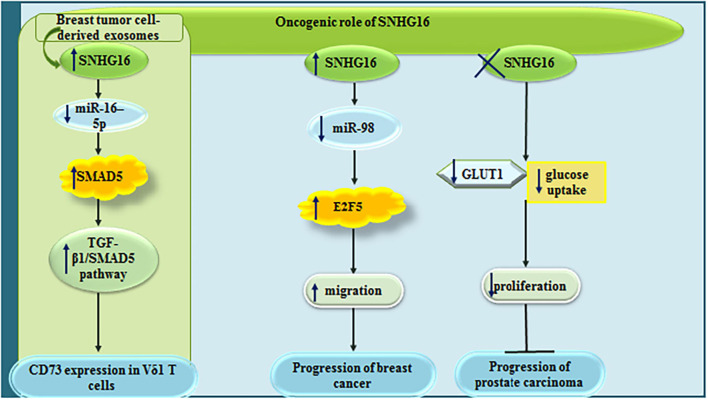
Oncogenic roles of SNHG6 in breast and prostate cancers.

Small nucleolar RNA host gene 6 has also important impacts on the modulation of tumor microenvironment through influencing function of γδ immunosuppressive T cells. Mechanistically, SNHG16 works as a sponge for miR-16-5p, thus augmenting expression of SMAD5 and potentiating the TGF-β1/SMAD5 pathway to increase expression of CD73 in Vδ1 T cells (Ni et al., 2020). In addition, SNHG16 can enhance migratory potential of breast cancer cells via sequestering miR-98 and releasing E2F5 from its inhibitory effects (Cai et al., 2017). In prostate cancer cells, siRNA-mediated silencing of SNHG16 results in down-regulation of GLUT-1, reduction of glucose uptake and inhibition of proliferation of cancerous cells without affecting normal prostate cells (Shao et al., 2020). Figure 2 shows the oncogenic roles of SNHG6 in breast and prostate cancers.

**FIGURE 3:**
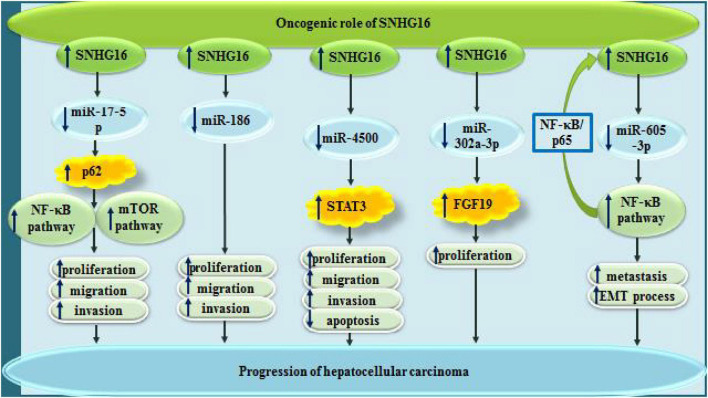
Oncogenic roles of SNHG16 in hepatocellular carcinoma via sponging miR-17-5p, miR-186, miR-4500, miR-302a-3p, and miR-605-3p.

**FIGURE 4:**
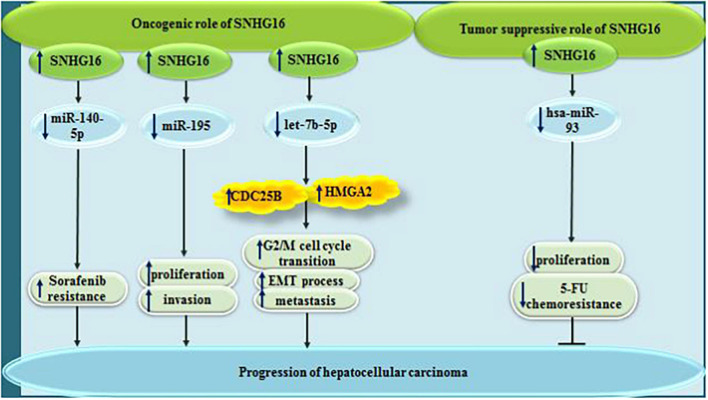
In hepatocellular carcinoma, while SNHG16 exerts oncogenic effect via sponging miR-140-5p, miR-195, and let7b-5p, it can have tumor suppressor effect via sponging has-miR-93.

In hepatocellular carcinoma (HCC), SNHG16 has diverse oncogenic as well as tumor suppressor roles (Figures 3, 4). SNHG16 has been shown to accelerate proliferation, migratory aptitude and invasiveness of HCC cells through sequestering miR-186 and enhancing expression of ROCK1 (Chen et al., 2019). Moreover, miR-4500 is another sponged miRNA by SNHG16 through which this lncRNA promotes development of HCC (Lin et al., 2019). In this type of cancer, SNHG16 also interacts with miR-302a-3p to increase expression of FGF19 and enhance cell proliferation (Li W. et al., 2019). Metastatic ability of HCC cells can be regulated by SNHG16 through sequestering miR-605-3p. This miRNA can suppress epithelial-mesenchymal transition (EMT) and metastatic ability of HCC via directly suppressing TRAF6 expression and further modulating NF-κB signaling. Being up-regulated by SNHG16, TRAF6 can in turn increase activity of SNHG16 promoter through activation of NF-κB, thus constructing an positive feedback loop in favor of HCC progression (Hu et al., 2020).

Contrary to the mentioned studies which reported the oncogenic effects of SNHG16 in the development of HCC, a single study has revealed down-regulation of SNHG16 in HCC cell lines. Ectopic virus-mediated over-expression of SNHG16 has repressed proliferation of HCC cells and attenuated their resistance to 5-FU through sponging hsa-miR-93 (Xu et al., 2018).

**FIGURE 5:**
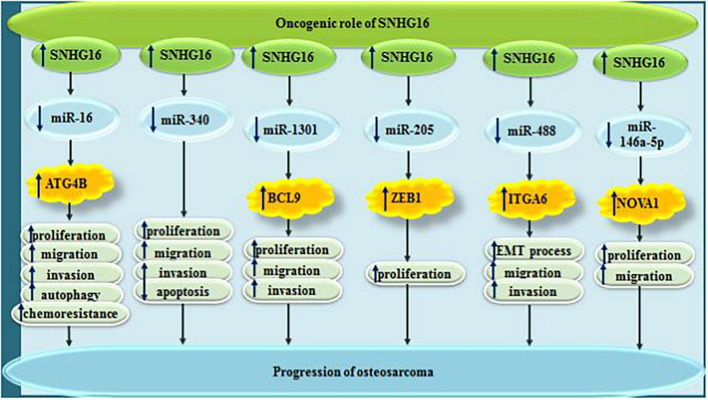
Oncogenic roles of SNHG16 in osteosarcoma.

In osteosarcoma, sponging impact of SNHG16 on miR-98-5p has an essential impact on proliferation, migration and invasive aptitude of cancer cell. Simultaneously, it can enhance cell cycle progression and decease cell apoptosis (Liao et al., 2019). Meanwhile, through sponging miR-16 and up-regulating ATG4B levels, SNHG16 can induce resistance to cisplatin in these cells (Liu Y. et al., 2019). SNHG16 can also promote proliferation of osteosarcoma cells through sponging miR-205 and enhancing expression of ZEB1 (Zhu C. et al., 2018). Finally, SNHG16 can facilitate EMT of osteosarcoma cells through miR-488/ITGA6 axis (Bu et al., 2021). Figure 5 depicts the oncogenic roles of SNHG16 in osteosarcoma.

Small nucleolar RNA host gene 6/miR-124-3p/MCP-1 has an important role in induction of cell proliferation and EMT in colorectal cancer (Chen et al., 2020). The sponging effect of SNHG16 on miR-200a-3p (Li Y. et al., 2019), miR-132-3p (He et al., 2020), and miR-302a-3p (Ke et al., 2019), also promotes tumorigenicity of colorectal cancer.

**FIGURE 6:**
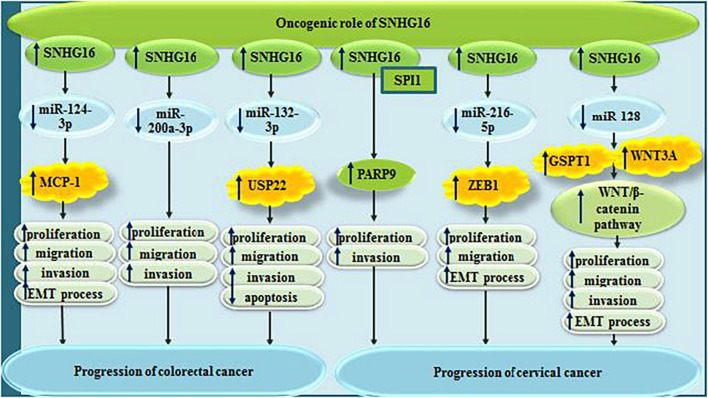
Oncogenic roles of SNHG16 in colorectal and cervical cancers.

In cervical cancer cells, SNHG16 has been found to recruit transcriptional factor SPI1 to increase expression of PARP9, thus promoting malignant behaviors of cells (Tao et al., 2020). Moreover, through sponging miR-216-5p, SNHG16 can increase expression of ZEB1, therefore increasing both cell proliferation and EMT process (Zhu H. et al., 2018). Finally, through sponging miR-128, it affects activity Wnt/β-catenin pathway (Wu et al., 2020). Figure 6 summarizes the role of SNHG16 in colorectal and cervical cancers.

**FIGURE 7:**
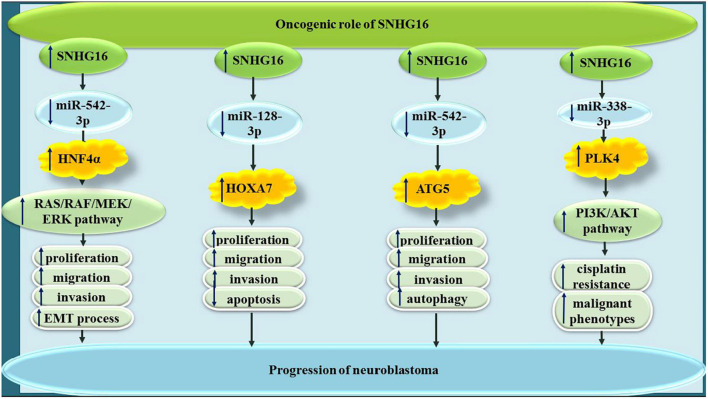
In neuroblastoma, SNHG16 has been revealed to sponge miR-542-3p, miR-128-3p, and miR-338-3p.

In neuroblastoma cells, SNHG16 has been revealed to sequester miR-542-3p (Deng et al., 2020), miR-128-3p (Bao et al., 2020) and miR-338-3p (Xu Z. et al., 2020), thus increasing expressions of HNF4α, HOXA7, and PLK4, respectively (Figure 7).

**FIGURE 8:**
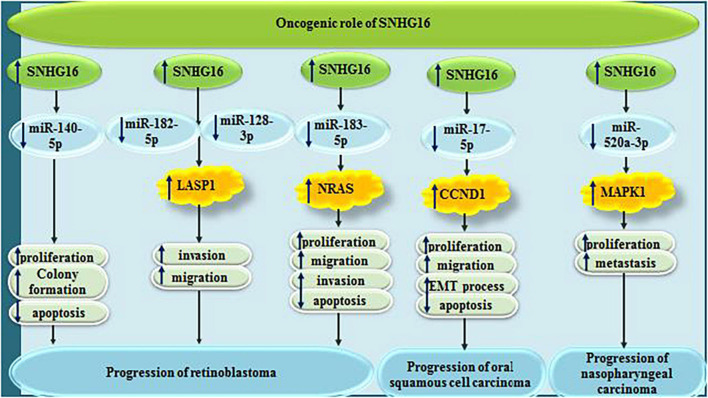
Oncogenic roles of SNHG16 in retinoblastoma, oral squamous cell carcinoma and nasopharyngeal carcinoma.

In other types of cancers, including retinoblastoma, oral squamous cell carcinoma, nasopharyngeal carcinoma, SNHG16 sequesters a number of miRNAs, namely miR-140-5p, miR-182-5p, miR-128-3p, miR-183-5p, miR-17-5p, and miR-520a-3p (Figure 8).

In pancreatic cancer, SNHG16 acts in favor of tumor progression through sponging miR-302b-3p and subsequently increasing expression of SLC2A4 (Xu et al., 2021). Moreover, it can contribute in this process through sponging miR-218-5p (Liu S. et al., 2019). Finally, SNHG16-mediated enhancement of lipogenesis through affecting expression of SREBP2 facilitates progression of pancreatic cancer (Yu et al., 2019b).

**FIGURE 9:**
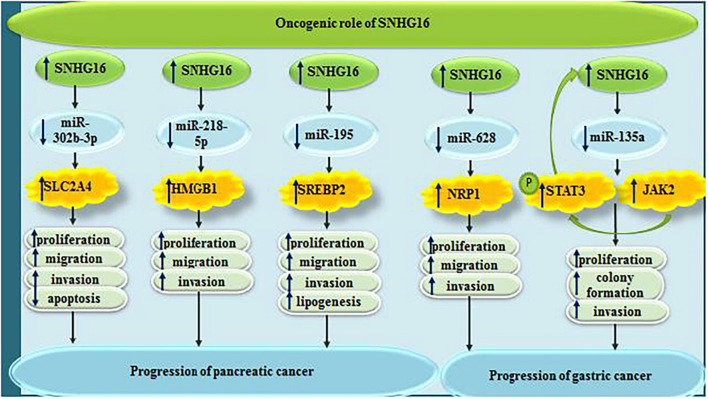
Oncogenic roles of SNHG16 in pancreatic and gastric cancers.

Small nucleolar RNA host gene 6 participates in the progression of gastric cancer via sequestering miR-628-3p and consequently decreasing expression of NRP1 (Pang et al., 2019). In this type of cancer, SNHG16 also sponges miR-135a and activates JAK2/STAT3 signaling (Wang et al., 2019b; Figure 9).

Table 1 summarizes the results of *in vitro* studies regarding the role of SNHG16 in carcinogenesis.

**TABLE 1:** Outline of researches which measured expression of SNHG16 in cell lines (Δ, knock-down or deletion; 5-FU, 5-fluorouracil; VM, vasculogenic mimicry).

**Tumor type**	**Interactions**	**Cell line**	**Function**	**References**
**Non−small cell lung cancer (NSCLC)**	miR-520a-3p, EphA2	16HBE, A549, NCI-H292, NCI-H460, NCI-H1703	Δ SNHG16: ↓ proliferation, ↓ migration, ↓ invasion, ↑ apoptosis	Yu et al., 2020
	miR-4500, USP21, YY1	A549, H1299, NCI-H460, and NCI-H520	Δ USP21: ↓ proliferation, ↓ migration, ↓ invasion	Xu P. et al., 2020
	miR-146a, MUC5AC	A549, NCI-H292, NCI-H460, NCI-H1703, 16HBE	Δ SNHG16: ↓ proliferation, ↓ migration, ↓ invasion ↑ SNHG16: ↑ proliferation, ↑ migration, ↑ invasion	Han et al., 2019
**Breast cancer**	miR-16–5p, SMAD5, TGF-β1/SMAD5 pathway, CD73	MCF-10A, MCF-7, T-47D, MDA-MB-231, HEK293T	_	Ni et al., 2020
	miR-98, E2F5	MDA-MB-231, MCF-7, MDA-MB468 and HEK293T	Δ SNHG16: ↓ migration, did not affect proliferation ↑ SNHG16: ↑ migration, did not affect proliferation	Cai et al., 2017
	let-7a-5p, RRM2	MCF-7	Δ SNHG16: ↓ proliferation	Zhong et al., 2019
**Prostate carcinoma**	GLUT1	22Rv1, HPrEC	Δ SNHG16: ↓ proliferation, ↓ glucose uptake	Shao et al., 2020
**Hepatocellular carcinoma (HCC)**	hsa-miR-93	Hep3B, HuH7, SNU398, SNU423, SNU429, Hep3G2, SK-HEP-1, and PLC/PRF/5	↑ SNHG16: ↓ proliferation, ↓ 5-FU chemoresistance	Xu et al., 2018
	miR-17-5p, p62, mTOR pathway, NF-κB pathway	Huh-7 and HepG2	Δ SNHG16: ↓ proliferation, ↓ migration, ↓ invasion ↑ SNHG16: ↑ proliferation, ↑ migration, ↑ invasion, ↑ cell cycle progression, ↓ apoptosis	Zhong et al., 2020
**Hepatocellular carcinoma (HCC)**	miR-186	Hep-3B, Huh7, Sk-hep-1, SMMC-7721, PLC, HL-77O2	Δ SNHG16: ↓ proliferation, ↓ migration, ↓ invasion	Chen et al., 2019
	miR-4500, STAT3	SMMC-7721, L02, MHCC-97H, HepG2	Δ SNHG16: ↓ proliferation, ↓ migration, ↓ invasion, ↑ apoptosis	Lin et al., 2019
	miR-302a-3p, FGF19	Huh7, HepG2, SMMC7721, SK-Hep1 and Hep 3B, LO2	Δ SNHG16: ↓ proliferation	Li W. et al., 2019
	miR-605-3p, NF-κB pathway	HCCLM3, MHCC97L, MHCC-97H, L02, Hep3B and HepG2	Δ SNHG16: ↓ metastasis, ↓ EMT process	Hu et al., 2020
	miR-140-5p	HepG2, SK-hep1, Huh7, and HCCLM3, LO2, HepG2/SOR	Δ SNHG16: ↓ sorafenib resistance	Ye et al., 2019
	miR-195	HepG2, SMMC7721, Hep3B, Bel7402, Huh7, LO2	Δ SNHG16: ↓ proliferation, ↓ invasion	Xie et al., 2019
	_	HL-7702, SK-Hep-1, Huh7, Hep3B, HepG2	Δ SNHG16: ↓ proliferation, ↓ migration, ↓ invasion, ↓ sorafenib resistance	Guo et al., 2019
	let-7b-5p, CDC25B, HMGA2	MHCC97H, HuH7, SMMC7721, Hep3B, HepG2, LO2, HEK293	Δ SNHG16: ↑ G2/M cell cycle arrest, ↓ cisplatin resistance, ↓ metastasis, ↓ EMT process	Li S. et al., 2020
**Osteosarcoma**	miR-98-5p	U2OS, Saos-2, HOS, MG-63, hFOB 1.19	Δ SNHG16: ↓ proliferation, ↓ migration, ↓ invasion, ↑ cell cycle arrest, ↑ apoptosis	Liao et al., 2019
	miR-16, ATG4B	SAOS2, U2OS, OB3, 293T	Δ SNHG16: ↓ proliferation, ↓ migration, ↓ invasion, ↓ autophagy, ↓ chemoresistance	Liu Y. et al., 2019
	miR-340	hFOB1.19, U2OS, SaOS2	Δ SNHG16: ↓ viability, ↓ invasion, ↑ apoptosis, ↑ caspase 3/7 activity	Su et al., 2019
	miR-1301, BCL9	U2OS, MG-63	Δ SNHG16: ↓ proliferation, ↓ migration, ↓ invasion	Wang et al., 2019a
	miR-205, ZEB1	MG-63, U2OS, SAOS2, HOS, OB3	Δ SNHG16: ↓ proliferation	Zhu C. et al., 2018
	miR-488, ITGA6	U2OS, HOS	Δ SNHG16: ↓ migration, ↓ invasion, ↓ EMT process	Bu et al., 2021
	miR-1285-3p, cleaved-caspase-3, Bax, pro-caspase-3, Bcl-2	U2OS, MNNG/HOS, 143b, SJSA, MG63, 293, hFOB 1.19	Δ SNHG16: ↓ proliferation, ↓ migration, ↓ invasion, ↑ cell cycle arrest, ↑ apoptosis	Xiao et al., 2021
	miR-146a-5p, NOVA1	hFOB1.19, MG63, U2OS, 143B, MNNG/HOS	↑ SNHG16: ↑ proliferation, ↑ migration	Zheng et al., 2019
**Colorectal cancer (CRC)**	miR-124-3p, MCP-1	HEK293T, FHC, SW480, HCT116, DLD-1, LOVO	Δ SNHG16: ↓ proliferation, ↓ migration, ↓ invasion, ↓ EMT process	Chen et al., 2020
	miR-200a-3p	CaCO-2, SW480, HCT116, LoVo, CCC-HIE-2	Δ SNHG16: ↓ proliferation, ↓ migration, ↓ invasion	Li Y. et al., 2019
	Wnt pathway, c-Myc, AGO, HuR, genes involved in lipid metabolism	HCT116, SW480, DLD1, 293T, K562, GM12878	Δ SNHG16: ↓ migration, ↑ apoptosis	Christensen et al., 2016
	miR-132-3p, USP22	SW480, SW620, CD841 CON	Δ SNHG16: ↓ proliferation, ↓ migration, ↓ invasion, ↑ apoptosis	He et al., 2020
	miR-302a-3p, AKT	HCT116, CaCO-2	Δ SNHG16: ↓ proliferation, ↑ SNHG16: ↑ proliferation	Ke et al., 2019
**Cervical cancer**	PARP9, SPI1	SiHa, CaSki, C33A, ME180, HeLa, HcerEpic	Δ SNHG16: ↓ proliferation, ↓ invasion	Tao et al., 2020
	miR-216-5p, ZEB1	HeLa, CaSki, SiHa, C33A, H8	Δ SNHG16: ↓ proliferation, ↓ migration	Zhu H. et al., 2018
	miR-128, GSPT1, WNT3A, WNT pathway	Endl/E6E7, HeLa, C33A	Δ SNHG16: ↓ proliferation, ↓ EMT process	
**Neuroblastoma (NB)**	_	SH-SY5Y	Δ SNHG16: ↓ proliferation, ↓ migration, ↑ G0/G1 phase arrest, ↑ apoptosis	Yu et al., 2019a
	miR-542-3p, HNF4α, RAS/RAF/MEK/ERK signaling pathway	SKNBE-2, SK-N-SH, HEK293, LAN-5	Δ SNHG16: ↓ proliferation, ↓ migration, ↓ invasion, ↓ EMT process	Deng et al., 2020
	miR-128-3p, HOXA7	SK-N-SH, IMR-32, SK-N-AS, SK-NDZ, HUVEC	Δ SNHG16: ↓ proliferation, ↓ migration, ↓ invasion, ↑ apoptosis	Bao et al., 2020
	miR-542-3p, ATG5	LAN1, SK-N-SH and IMR-32, HUVEC	Δ SNHG16: ↓ proliferation, ↓ migration, ↓ invasion, ↓ autophagy	Wen et al., 2020
	miR-338-3p, PLK4, PI3K/AKT pathway	SK-N-AS, SK-N-SH, SK-N-AS-R and SK-N-SH-R	Δ SNHG16: ↓ cisplatin resistance, ↓ malignant phenotypes	Xu Z. et al., 2020
**Retinoblastoma (RB)**	miR-140-5p	ARPE-19, WERI Rb1, SO-RB-50, Y79, SO-Rb50	Δ SNHG16: ↓ proliferation, ↓ colony formation, ↑ apoptosis	Xu et al., 2019
	miR-182-5p, miR-128-3p, LASP1	WERI-RB1, SO-RB50, Y79, ARPE-19	Δ SNHG16: ↓ migration, ↓ invasion	Yang L. et al., 2019
	miR-183-5p, NRAS	ARPE-19 and human RB cell lines Y-79, WERI-Rb-1, 67BR and SO-Rb50	Δ SNHG16: ↓ proliferation, ↓ migration, ↓ invasion, ↑ apoptosis	Sun et al., 2019
**Oral squamous cell carcinoma (OSCC)**	c-Myc, E-cadherin, N-cadherin, Snail, MMP-2, MMP-9, PCNA	SCC-25, CAL-27, NHOK, Tca8113, TSCCA	Δ SNHG16: ↓ proliferation, ↓ migration, ↓ invasion, ↓ EMT process, ↑ apoptosis	Li S. et al., 2019
	miR-17-5p, CCND1, N-cadherin, Vimentin	NOK, CAL27, TCA8113, OEC-M1, TW2.6	Δ SNHG16: ↓ proliferation, ↑ apoptosis ↑ SNHG16: ↑ proliferation, ↑ migration, ↑ EMT process	Wang et al., 2021
**Pancreatic cancer (PC)**	miR-302b-3p, SLC2A4	HPY-Y5, BxPC3, Panc-1, MIA Paca-2, SW1990	Δ SNHG16: ↓ proliferation, ↓ migration, ↓ invasion, ↑ apoptosis	Xu et al., 2021
	miR-218-5p, HMGB1	BxPC-3, SW1990, PANC-1, AsPC1, HPDE6-C7	Δ SNHG16: ↓ proliferation, ↓ colony formation, ↓ migration, ↓ invasion	Liu S. et al., 2019
	miR-195, SREBP2	HPDE6-C7, PANC-1, AsPC-1, BxPC-3, SW1990, HEK-293	Δ SNHG16: ↓ proliferation, ↓ migration, ↓ invasion, ↓ lipogenesis	Yu et al., 2019b
**Nasopharyngeal carcinoma (NPC)**	miR-520a-3p, MAPK1	SUNE1, 5–8F, C666-1, NP69	Δ SNHG16: ↓ proliferation, ↓ metastasis	Wu et al., 2021
**Gastric cancer**	miR-628, NRP1	BGC-823, SGC-7901, MKN-45, AGS, GES-1	Δ SNHG16: ↓ proliferation, ↓ migration, ↓ invasion	Pang et al., 2019
	miR-135a, JAK2/STAT3 pathway	BGC823, MGC803, MKN45, SGC7901, GES-1	Δ SNHG16: ↓ proliferation, ↓ colony formation, ↓ invasion	Wang et al., 2019b
**Papillary thyroid cancer (PTC)**	miR-497, BDNF, YAP1	IHH-4, TPC-1, HTH83, Nthy-ori 3-1	Δ SNHG16: ↓ proliferation, ↓ migration, ↓ invasion, ↑ apoptosis	Wen et al., 2019
**Bladder cancer (BC)**	p21	T-24, BIU87, 5637, SV-HUC-1	Δ SNHG16: ↓ proliferation, ↓ colony formation, ↑, G1 phase arrest, ↑ apoptosis	Cao et al., 2018
	miR-17-5p, TIMP3	5637, J82, RT4, T24	Δ SNHG16: ↓ proliferation, ↓ viability, ↓ EMT process, ↑ apoptosis	Peng and Li, 2019
**Ovarian cancer**	P-AKT, MMP9	SKOV-3, ES2, HO8910, OMC685, OSE-29	Δ SNHG16: ↓ proliferation, ↓ migration, ↓ invasion	Yang et al., 2018
**Acute myeloid leukemia (AML)**	miR183-5p, FOXO1	THP1, HL60, Kasumi 3, AML139, PBMCs	Δ SNHG16: ↓ proliferation, ↑ G0/G1-phase arrest, ↑ apoptosis	Yang R. et al., 2020
	CELF2, PTEN, PI3K/AKT signaling	HS-5, HL60, BDCM, AML-193, Kasumi-6	Δ SNHG16: ↓ proliferation, ↓ migration ↑ SNHG16: ↑ proliferation, ↑ migration	Shi et al., 2021
**Leukemia**	miR-193a-5p, CDK8	Kasumi-1, KG-1, MV-4-11, THP-1, K-562, HL-60, RPMI-1788	Δ SNHG16: ↓ proliferation, ↓ viability, ↑ apoptosis	Piao and Zhang, 2020
**Acute lymphoblastic leukemia**	miR-124-3p,	MOLT3, MOLT4, SUP-B15, CCRF-CEM, RS4;11, TALL104, CEM/C1, CEM/C2, Loucy, BMMC, PBMC	↑ SNHG16: ↑ proliferation, ↑ migration	Yang T. et al., 2019
**Large B-cell lymphoma**	miR-497-5p, PIM1	OCI-LY7, OCI-LY3	Δ SNHG16: ↓ proliferation, ↑ G0/G1 phase arrest, ↑ apoptosis	Zhu et al., 2019
**Multiple myeloma**	miR-342-3p	RPMI-8226, NCI-H929	Δ SNHG16: ↓ proliferation	Yang X. et al., 2020
**Glioma**	miR-373, EGFR, PI3K/AKT pathway	NHAs, U251, LN229, U87	Δ SNHG16: ↓ proliferation, ↓ migration, ↓ invasion	Zhou et al., 2020
**Glioma**	miR-490, PCBP2	T98G, U251, NHA	Δ SNHG16: ↓ proliferation, ↓ migration, ↓ invasion	Kong et al., 2020
	miR-4518, PRMT5, Bcl-2, PI3K/Akt pathway,	NHAs, U251, H4, SW1783, LN229	Δ SNHG16: ↓ proliferation, ↑ apoptosis	Lu et al., 2018
	miR-212-3p, USF1, ALDH1A1	HA, U87, U251, HEK293T	Δ SNHG16: ↓ proliferation, ↓ migration, ↓ invasion, ↓ VM	Wang et al., 2019c
	miR-424-5p,	T98G, LN229	↑ SNHG16: ↓ effect of Ropivacaine, ↑ proliferation, ↑migration, ↑ invasion, ↓ apoptosis	Liu et al., 2020
	TLR7, NFκB/c-Myc signaling, MyD88	SHG44, U251	Δ SNHG16: ↓ proliferation, ↓ migration, ↓ invasion ↑ SNHG16: ↑ proliferation,↑ migration, ↑ invasion	Zhang et al., 2021
**Endometrial carcinoma**	miR-490-3p, TFAP2A, HK2	HEC-1B, HEC-1A, RL95-2, AN3CA, EMC	Δ SNHG16: ↓ proliferation, ↓ glycolysis	Zhang G. et al., 2019
**Laryngeal squamous cell carcinoma**	miR-877-5p, FOXP4	16HBE, AMC-HN-8	Δ SNHG16: ↓ proliferation, ↓ migration, ↓ invasion	Wang et al., 2020
**Esophageal cancer**	Wnt/β-catenin pathway	TE-13, TE-1, EC-1, Eca-109, HEEC	Δ SNHG16: ↓ proliferation, ↓ invasion, ↑ apoptosis	Han et al., 2018
	miR-140-5p, ZEB1	eca109, EC9706, TE1, Kyse-30, Kyse-70, HEEC	Δ SNHG16: ↓ proliferation, ↓ migration, ↓ EMT process, ↑ apoptosis	Zhang et al., 2018
**Hemangioma (HA)**	miR-520d-3p, STAT3	HemECs	ΔSNHG16: ↓ proliferation, ↓ migration, ↓ invasion, ↓ vasoformation, ↑ apoptosis	Zhao et al., 2018

## Animal Studies

Animal studies have consistently shown that SNHG16 silencing decreases malignant feature of the grafted cancer cells (Table 2). The only exception has been reported in HCC where SNHG16 over-expression has significantly suppressed the *in vivo* expansion of grafted HuH7 cells (Xu et al., 2018). Another study in HCC xenograft model has shown that SNHG16 silencing enhances response of HepG2/SOR cells to cytotoxic effect of sorafenib and attenuates tumor growth (Ye et al., 2019). In xenograft models of retinoblastoma, up-regulation SNHG16 (Xu et al., 2019) or its downstream target NRAS (Sun et al., 2019) can increase tumor growth. Finally, in gastric cancer where SNHG16 sponges miR-628, *in vivo* studies have shown that up-regulation of miR-628 can decrease tumor expansion (Pang et al., 2019).

**TABLE 2:** Outline of studies which judged function of SNHG16 in animal models (Δ, knock-down or deletion; VM, vasculogenic mimicry).

**Tumor Type**	**Animal models**	**Results**	**References**
**Non−small cell lung cancer (NSCLC)**	male Athymic BALB/c mice	Δ SNHG16: ↓ tumor volume, ↓ tumor weight, ↓ tumor growth	Yu et al., 2020
	male Athymic BALB/c mice	Δ SNHG16: ↓ tumor volume, ↓ tumor weight	Han et al., 2019
**Hepatocellular carcinoma (HCC)**	athymic nude mice	↑ SNHG16: ↓ tumorigenicity	Xu et al., 2018
	male athymic nude mice	Δ SNHG16: ↓ tumor size, ↓ tumor weight ↑ SNHG16: ↑ tumor size, ↑ tumor weight	Zhong et al., 2020
	female BALB/c nude mice	Δ SNHG16: ↓ tumor volume, ↓ tumor weight, ↓ tumor growth ↑ SNHG16:↑ tumor volume, ↓ tumor growth	Chen et al., 2019
	nude mice	Δ SNHG16: ↓ number and size of metastatic colonies, ↓ tumor weight, ↓ tumor growth	Hu et al., 2020
	Male Athymic nu/nu nude mice	Δ SNHG16: ↓ tumor size, ↓ tumor weight, ↓ tumor growth, ↓ sorafenib resistance	Ye et al., 2019
	male BALB/c nude mice	Δ SNHG16: ↓ tumor weight, ↓ tumor growth	Xie et al., 2019
	BALB/c nude mice	Δ SNHG16: ↓ tumor volume, ↓ tumor weight, ↓ metastatic	Li S. et al., 2020
**Osteosarcoma**	male BALB/c mice	Δ SNHG16: ↓ tumor volume, ↓ EMT process, ↓ tumor growth, ↓ metastasis	Bu et al., 2021
	male BALB/c nude mice	Δ SNHG16: ↓ tumor volume, ↓ tumor weight	Xiao et al., 2021
**Colorectal cancer (CRC)**	nude mice	Δ SNHG16: ↓ tumor size, ↓ tumor weight, ↓ metastasis	Chen et al., 2020
	male BALB/c nude mice	↑ SNHG16: ↑ tumor size	Li Y. et al., 2019
	male BALB/c-nude mice	Δ SNHG16: ↓ tumor weight, ↓ metastasis, ↓ tumor growth	He et al., 2020
**Cervical cancer**	specific-pathogen-free BALB/c-nu/nu nude mice	Δ SNHG16: ↓ tumor growth	Tao et al., 2020
**Neuroblastoma (NB)**	BALB/c nude mice	Δ SNHG16: ↓ tumor volume, ↓ tumor weight	Deng et al., 2020; Bao et al., 2020, Wen et al., 2020
	athymic BALB/c mice	Δ SNHG16: ↓ tumor volume, ↓ tumor weight	Xu Z. et al., 2020
**Retinoblastoma (RB)**	male BALB/c nude mice	Δ SNHG16: ↓ tumor volume, ↓ tumor weight	Xu et al., 2019
	female BALB/c nude mice	Δ NRAS: ↓ tumor volume, ↓ tumor weight	Sun et al., 2019
**Oral squamous cell carcinoma**	BALB/c-nude mice	Δ SNHG16: ↓ tumor volume, ↓ tumor weight	Li S. et al., 2019
	male athymic BALB/c nude mice	Δ SNHG16: ↓ tumor growth ↑SNHG16: ↑ tumor growth	Wang et al., 2021
**Pancreatic cancer**	male BALB/c nude mice	Δ SNHG16: ↓ tumor volume, ↓ tumor growth	Liu S. et al., 2019
**Nasopharyngeal carcinoma (NPC)**	male BALB/C nude mice	Δ SNHG16: ↓ tumor volume, ↓ tumor weight	Wu et al., 2021
**Gastric cancer**	female BALB/c nude mice	↑ miR-628: ↓ tumor volume, ↓ tumor weight	Pang et al., 2019
**Acute lymphoblastic leukemia (ALL)**	null mice	Δ SNHG16: ↓ tumor volume, ↓ ALL tumor transplants	Yang T. et al., 2019
**Large B−cell lymphoma (DLBCL)**	male NOD/SCID mice	Δ SNHG16: ↓ tumor growth	Zhu et al., 2019
**Glioma**	athymic BALB/c nude mice	Δ SNHG16: ↓ tumor volume, ↓ number of VMs, ↑ survival period	Wang et al., 2019c
**Endometrial carcinoma**	male nude BALB/c mice	Δ SNHG16: ↓ tumor volume, ↓ tumor growth	Zhang G. et al., 2019
**Laryngeal squamous cell carcinoma (LSCC)**	female nude mice	Δ SNHG16: ↓ tumor volume, ↓ tumor weight	Wang et al., 2020
**Esophageal cancer**	female BALB/c athymic nude mice	Δ SNHG16: ↓ tumor growth	Han et al., 2018

## Clinical Studies

Except for a single study which demonstrated down-regulation of SNHG16 in HCC samples versus nearby non-malignant hepatic tissues (Xu et al., 2018), other studies have indicated up-regulation of SNHG16 in malignant tissues of different origins compared with non-neoplastic samples (Supplementary Table 1). Consistent with these findings, up-regulation of SNHG16 has been revealed to predict poor survival of patients. Moreover, its expression has been related with greater chance of distant metastasis, lymph node involvement and low differentiation of tumor cells.

## Discussion

Small nucleolar RNA host gene 6 has been regarded as an oncogenic lncRNA in almost all tissues. This lncRNA affect carcinogenesis through multifaceted mechanisms including mechanisms related to both tumor cells and their niche. In fact, it can both affect cellular functions and processes, particularly those related with proliferation, survival and apoptosis as well as microenvironmental aspects of cancer progression.

More than 20 miRNAs have been found to interact with SNHG16. The sponging effects of SNHG16 on miRNAs have been well studied. miR-520a-3p, miR-4500, miR-146a miR-16–5p, miR-98, let-7a-5p, hsa-miR-93, miR-17-5p, miR-186, miR-302a-3p, miR-605-3p, miR-140-5p, miR-195, let-7b-5p, miR-16, miR-340, miR-1301, miR-205, miR-488, miR-1285-3p, miR-146a-5p, and miR-124-3p are examples of miRNAs sponged by this lncRNA in different types of cancers. Verification of interaction between this lncRNA and a number of miRNAs such as miR-98 in different tissues raises the possibility of independence of such interactions from the tissue type. TGF-β1/SMAD5, mTOR, NF-κB, RAS/RAF/MEK/ERK, PI3K/AKT, and Wnt/β-catenin pathways are among cancer-related pathways being affected by this lncRNA. Moreover, SNHG16 has been shown to affect expression of a number of EMT-associated transcription factors and enhance this process. SNHG16 has also been found to affect response of cancer cells to 5-FU and sorafenib.

Based on the results of functional studies that confirmed the ability of siRNA-mediated SNHG16 silencing in reduction of cancer cell proliferation and invasiveness, this strategy can be proposed as a therapeutic strategy for cancer. *In vivo* studies have also confirmed applicability of these methods; however no clinical study has applied these methods yet. Antisense oligonucleotides as a promising strategy for suppression of expression of SNHG16 should be appraised in clinical settings considering the bioavailability and safety issues.

Although over-expression of SNHG16 has been verified in tissue samples of different types of tumors, application of this lncRNA as a circulatory marker for early detection of cancer has not been assessed. Since clinical studies have revealed correlation between expression amounts of SNHG16 and malignant features, one can suppose that SNHG16 can be used as both diagnostic and prognostic marker. However, this speculation should be verified in future.

## Author Contributions

MT and SG-F wrote the draft and revised it. TK and SS collected the data and designed the tables and figures. All authors read and approved submitted version.

## Conflict of Interest

The authors declare that the research was conducted in the absence of any commercial or financial relationships that could be construed as a potential conflict of interest.

## Publisher’s Note

All claims expressed in this article are solely those of the authors and do not necessarily represent those of their affiliated organizations, or those of the publisher, the editors and the reviewers. Any product that may be evaluated in this article, or claim that may be made by its manufacturer, is not guaranteed or endorsed by the publisher.

## Footnotes

1
http://asia.ensembl.org/
