# Supplementary material for: A Review on the Role of Small Nucleolar RNA Host Gene 6 Long Non-coding RNAs in the Carcinogenic Processes
Source: Front Cell Dev Biol. 2021 Oct 4;9:741684. doi: 10.3389/fcell.2021.741684 (PMC8522957; doi:10.3389/fcell.2021.741684)
Supplement: Supplementary file 1 [file Table_1.docx]

**Supplementary Table S1**. Results of papers that described dysregulation of SNHG16 in clinical samples (ANCTs: adjacent non-cancerous tissues, OS: Overall survival, DFS: Disease-free survival, PFS: progression free survival, TNM: tumor‐node‐metastasis, ALT: alanine aminotransferase, PVTT: portal vein tumor thrombus, GEO: Gene Expression Omnibus, MSS: microsatellite stable, MSI: microsatellite instable, FIGO: International Federation of Gynecology and Obstetrics, ATCC: American Type Culture Collection, CIN: cervical intraepithelial neoplasia, INSS: international neuroblastoma staging system, MYCN: V-myc myelocytomatosis viral-related oncogene, neuroblastoma derived, PTBE: peritumoral brain edema, NBTs: normal brain tissues, WHO: World Health Organization, RLH: reactive lymph node hyperplasia, RFS: Recurrence free survival, ESCC: Esophageal squamous cell carcinoma).

| Tumor type | Samples | Expression  (Tumor vs. Normal) | Kaplan-Meier analysis (impact of SNHG16 up-regulation) | Univariate/ Multivariate cox regression | Clinicopathologic characteristics of patients | Reference |
| --- | --- | --- | --- | --- | --- | --- |
| Non‐small cell lung cancer (NSCLC) | 30 Tumor specimens and ANCTs | high | _ | _ | _ | (1) |
|  | 66 Lung cancer tissues and ANCTs | high | shorter DFS and OS | SNHG16 expression and TNM stage were independent predictors for DFS and OS. | SNHG16 level was associated with tumor size, TNM stage and lymph node metastasis. | (3) |
| Breast cancer | microarray analysis | high | _ | _ | _ | (4) |
|  | GEO analysis: 45 tumor samples and 5 nontumorous samples (GSE36295) | high | _ | _ | _ | (31) |
|  | GEO analysis: 104 tumor samples and 17 nontumorous samples  (GSE42568) |  |  |  |  |  |
|  | GEO analysis: 25 tumor samples and 25 nontumorous samples  (GSE109169) |  |  |  |  |  |
|  | TCGA analysis |  |  |  |  |  |
| Prostate carcinoma | 52 prostate carcinoma patients and 36 normal controls | high | _ | _ | _ | (6) |
| Hepatocellular carcinoma (HCC) | 43 pairs of HCC tissues and ANCTs | down | _ | _ | _ | (11) |
|  | 3 HCC samples  and 3 hepatic hemangiomas | high | _ | _ | _ | (32) |
|  | 30 control-matched  HCC tissues | high | _ | _ | _ |  |
|  | 108 HCC patient tissues | high | shorter DFS and OS | High SNHG16 expression, multiple tumors, macrovascular invasion, and alpha fetoprotein ≥ 400 ng/ml could be regarded as independent predictors for OS and DFS in HCC patients. | Patients who had higher expression of SNHG16 indicated higher chance of multiple tumors and macrovascular invasion. |  |
|  | 50 cases of HCC tissues and matched ANCTs | high | _ | _ | Chi-square test: The SNHG16 level was associated with tumor size, TNM stage; ALT level and HBV DNA level. | (7) |
|  | 88 pairs of HCC tissues and ANCTs | high | shorter OS | TNM stage and SNHG16 expression was found to be as independent prognostic factors of HCC. | SNHG16 expression was associated with tumor size, lymph node status, and TNM stage. | (8) |
|  | 34 HCC tissue samples and normal liver tissues | high | shorter DFS and OS | _ | _ | (9) |
|  | 78 HCC tumor samples and matched ANCTs | high | _ | _ | SNHG16 expression was associated with tumor size, tumor thrombus, presence of an envelope and tumor satellites. | (10) |
|  | 103 HCC tissues and corresponding  ANCTs | high | _ | _ | SNHG16 expression was associated with tumor size, TNM stage, and vascular invasion. | (33) |
| Hepatocellular carcinoma (HCC) | 40 pairs of HCC tissues and ANCTs | high | _ | _ | SNHG16 expression was associated with TNM stage, and metastasis. | (34) |
|  | 10 fresh HCC tissues and ANCTs | high | _ | _ | _ | (35) |
|  | 61 formalin-fixed, paraffin-embedded samples from HCC patients | high | shorter DFS and OS | SNHG16 expression, AFP level, PVTT, and metastasis were found to be significant independent predictors for HCC patients. | SNHG16 expression was associated with tumor size, AFP level, PVTT, and metastasis. |  |
|  | 47 pairs of HCC tissues and ANCTs | high | shorter DFS and OS | _ | _ | (36) |
| Osteosarcoma | 96 pairs of OS tissues and ANCTs | high | shorter OS | SNHG16 expression and TNM stage were found to be independent prognostic factors for osteosarcoma patients | SNHG16 expression was strongly correlated with tumor size, TNM stage, and metastasis and advanced stage. | (12) |
|  | 30 pairs of cancer tissues and ANCTs | high | _ | _ | _ | (13) |
|  | 25 pairs of cancer tissues and ANCTs | high | _ | _ | _ | (37) |
|  | 65 pairs of cancer and ANCTs | high | shorter OS | _ | SNHG16 expression was positively associated with advanced tumor stages, larger tumor size and higher chance of distance metastasis. | (38) |
|  | 20 pairs of cancer and ANCTs | high | _ | _ | _ | (14) |
|  | 10 pairs of cancer tissues and ANCTs | high | _ | _ | _ | (15) |
|  | 50 pairs of cancer tissues and ANCTs |  | poor prognosis |  | SNHG16 expression was associated with clinical stage. | (39) |
|  | cancer tissues and ANCTs | high | poor survival | _ | SNHG16 expression was associated with tumor stage of OS. | (40) |
| Colorectal cancer (CRC) | 120 CRC and ANCTs | high | poor survival | _ | SNHG16 expression was associated with tumor grade. | (16) |
|  | 56 pairs of CRC tissues and ANCTs | high | shorter OS | - | SNHG16 expression was associated with metastasis, and lymph node. | (17) |
|  | 281 fresh frozen MSS or MSI, primary stage I–IV CRCs, 33 adenomas and 292 ANCTs | high | _ | _ | _ | (41) |
|  | 20 normal mucosa, 39 adenomas and 44 adenocarcinomas | high | _ | _ | _ |  |
|  | 50 pairs of CRC tissues and ANCTs | high | _ | _ | _ | (18) |
| Cervical Cancer | 66 cervical cancer samples and 20 normal cervical samples | high | shorter survival | _ | SNHG16 expression was closely associated with size of tumors, differentiation degree, and FIGO classification. | (20) |
| Cervical cancer | 38 cervical tumor samples, 22 CIN samples, and 18 normal controls | high | shorter OS | High expression level of SNHG16 was found to be an independent prognostic factor. | SNHG16 expression was associated with greater tumor size, advanced FIGO stage, lymph node metastasis, and poor differentiation. | (21) |
|  | ATCC analysis: 411 CC patient tissues and 19 normal tissues | high | _ | _ | _ | (22) |
|  | 48 pairs of CC tissues and ANCTs | high | shorter OS | _ | SNHG16 expression was associated with TNM stage, tumor size, and distant metastasis. |  |
| Neuroblastoma (NB) | GEO analysis: 498 samples (GSE62564) | high | worse event-free survival and OS | _ | _ | (42) |
|  | 51 samples (GSE16237) | high | _ | _ | _ |  |
|  | 40 NB patients | high | _ | _ | SNHG16 expression was associated with clinical staging of NB tumor progression. |  |
|  | 30 pairs of NB tissues and ANCTs | high | _ | _ | _ | (23) |
|  | 48 NB tissues and 38 adjacent normal tissues | high | _ | _ | _ | (24) |
|  | 45 paired NB tissues and corresponding non-tumors samples | high | shorted OS | _ | SNHG16 expression was associated with INSS stage and MYCN status. | (43) |
|  | 76 neuroblastoma tissues | Higher in cisplatin-resistant group than cisplatin-sensitive group | shorted median survival time | _ | SNHG16 expression was associated with INSS staging and metastasis. | (25) |
| Retinoblastoma (RB) | 30 RB tissues and 10 normal retinas | high | _ | _ | _ | (44) |
|  | 76 human retinoblastoma specimens and 15 normal retina tissues | high | poor OS | _ | SNHG16 expression was associated with TNM stage, choroidal invasion, and optic nerve invasion. | (45) |
| Oral squamous cell carcinoma (OSCC) | 29 pairs of OSCC tissues and normal tissues | high | _ | _ | _ | (47) |
|  | 50 pairs of OSCC tissues, and their corresponding ANCTs | high | _ | _ | _ | (48) |
| Pancreatic cancer (PC) | 30 pairs of PC tissues, and their corresponding ANCTs | high | shorter OS | _ | _ | (26) |
|  | 46 pairs of PC tissues and ANCTs | high | lower survival | _ | SNHG16 expression was closely associated with poor differentiation, advanced TNM stage and lymph node metastasis. | (27) |
| Nasopharyngeal carcinoma (NPC) | GEO analysis: (GSE12452) | high | _ | _ | _ | (49) |
|  | 26 NPC tissues and ANCTs | high | lower survival time | _ | SNHG16 expression was correlated with clinical stage and lymph node metastasis. |  |
| Gastric cancer | 32 pairs of GC tissues and corresponding non-tumor normal tissues | high | poorer survival time | _ | SNHG16 expression was correlated with tumor size and TNM staging. | (30) |
| Papillary thyroid cancer (PTC) | 48 pairs of PTC tissues and ANCTs | high | _ | _ | SNHG16 expression was correlated with TNM stage and lymph node metastasis. | (50) |
| Bladder cancer (BC) | 46 BC tissues and matched ANCTs | high | shorter OS | _ | SNHG16 expression was correlated with metastasis, lymph node and pathological stage. | (51) |
|  | 275 BC tissues and ANCTs | high | _ | Patients with higher histological grade or more advanced tumor stage had significantly elevated odds of poor prognosis | SNHG16 expression was correlated with tumor grade and metastatic lymph nodes. | (52) |
|  | 10 BC patients and 100 healthy controls | high | _ | _ | _ | (71) |
|  | 120 BC patients, 68 benign disease and 52 healthy samples | high | _ | _ | _ | (72) |
|  | 100 BC patients, 52 benign disease and 48 healthy samples |  |  |  |  |  |
| Ovarian cancer | 103 pairs of ovarian cancer tissues and ANCTs | high | poor prognosis | _ | SNHG16 expression was associated with clinical stage, tumor size, lymph node metastasis and distant metastasis | (53) |
| Acute myeloid leukemia (AML) | peripheral blood samples from 76 AML patients and 68 healthy controls | high | _ | _ | _ | (54) |
|  | bone marrow blood specimenss from 30 AML patients and 30 controls | high | _ | _ | _ | (55) |
| Acute lymphoblastic leukemia (ALL) | 37 patients with ALL and 43 healthy controls | high | _ | _ | _ | (57) |
| Large B‐cell lymphoma (DLBCL) | 48 DLBCL tissues and 14 RLH control tissues | high | _ | _ | SNHG16 expression was positively associated with tumor stage. | (58) |
| Multiple myeloma (MM) | 20 primary MM patients and 15 marrow healthy samples | high | _ | _ | _ | (59) |
| Glioma | 40 glioma tissues and 10 normal brain tissues | high | _ | _ | SNHG16 expression was positively associated with tumor stage. | (60) |
|  | 31 glioma tissues and ANCTs | high | _ | _ | _ | (61) |
|  | 48 pairs of brain glioma tissues and PTBE tissues | Higher in glioma tissues than PTBE tissues | poorer OS and PFS | SNHG16 expression was found to be an independent prognostic indicator for the OS and PFS of patients with glioma. | SNHG16 expression was associated with larger tumor size and advanced WHO stage. | (62) |
|  | 5 glioma tissues and NBTs | high | _ | _ | _ | (63) |
|  | 30 cancerous tissues and normal para-carcinoma tissues | high | shorter 7-year survival rate | _ | SNHG16 expression was associated with the tumor grade. | (65) |
| Endometrial carcinoma |  | high | lower OS and RFS | _ | _ | (66) |
| Laryngeal squamous cell carcinoma (LSCC) | 35 LSCC tissues and normal tissues | high | _ | _ | SNHG16 expression was associated with clinical stage and lymph node metastasis | (67) |
| Esophageal cancer | 128 ESCC tissues and matched ANCTs | high | shorter OS | SNHG16 expression was found to be an independent predictor for overall survival of ESCC patients. | SNHG16 expression was associated with tumor stage, lymph nodes metastasis and clinical stage. | (68) |
|  | 68 pairs of ESCC tissues and ANCTs | high | _ | _ | _ | (69) |
| Hemangioma (HA) | 12 normal skin tissues, 12 involuting phase HA tissues, and 12 proliferating phase HA samples | higher in proliferating phase HA tissues | _ | _ | _ | (70) |
